# New Method for Tomato Disease Detection Based on Image Segmentation and Cycle-GAN Enhancement

**DOI:** 10.3390/s24206692

**Published:** 2024-10-17

**Authors:** Anjun Yu, Yonghua Xiong, Zirong Lv, Peng Wang, Jinhua She, Longsheng Wei

**Affiliations:** 1 Jiangxi Ganyue Expressway Co., Ltd., Nanchang 330200, China; yaj@600269.cn; 2 School of Automation, China University of Geosciences, Wuhan 430074, China; 1202111346@cug.edu.cn (Z.L.); wp15886382059@163.com (P.W.); weilongsheng@163.com (L.W.); 3 Hubei Key Laboratory of Advanced Control and Intelligent Automation for Complex Systems, Wuhan 430074, China; 4 Engineering Research Center of Intelligent Technology for Geo-Exploration, Ministry of Education, Wuhan 430074, China; 5 School of Engineering, Tokyo University of Technology, Tokyo 192-0982, Japan; she@stf.teu.ac.jp

**Keywords:** image segmentation, deep-learning, image enhancement, Cycle-GAN, plant disease detection

## Abstract

A major concern in data-driven deep learning (DL) is how to maximize the capability of a model for limited datasets. The lack of high-performance datasets limits intelligent agriculture development. Recent studies have shown that image enhancement techniques can alleviate the limitations of datasets on model performance. Existing image enhancement algorithms mainly perform in the same category and generate highly correlated samples. Directly using authentic images to expand the dataset, the environmental noise in the image will seriously affect the model’s accuracy. Hence, this paper designs an automatic leaf segmentation algorithm (AISG) based on the EISeg segmentation method, separating the leaf information with disease spot characteristics from the background noise in the picture. This algorithm enhances the network model’s ability to extract disease features. In addition, the Cycle-GAN network is used for minor sample data enhancement to realize cross-category image transformation. Then, MobileNet was trained by transfer learning on an enhanced dataset. The experimental results reveal that the proposed method achieves a classification accuracy of 98.61% for the ten types of tomato diseases, surpassing the performance of other existing methods. Our method is beneficial in solving the problems of low accuracy and insufficient training data in tomato disease detection. This method can also provide a reference for the detection of other types of plant diseases.

## 1. Introduction

With the deepening of agricultural mechanization, people can produce enough economic crops to meet daily needs. However, the production safety of crops is still threatened by many factors such as climate change, plant diseases, etc. [1]. Plant diseases, in particular, can seriously affect the agricultural economy, with mild cases affecting only a few plants and severe cases resulting in a significant decrease in crop yield across entire fields [2]. Traditional methods for detecting plant diseases rely on on-site judgments by agricultural experts or identification by farmers based on their own experience. Conventional subjective methods are burdensome and require significant labor, while inexperienced farmers may make misjudgments and indiscriminately use pesticides, which leads to the escalation of disease spread. Environmental factors and planting methods also easily interfere with growers’ recognition of diseases. To tackle these challenges, the use of deep learning technology for automatic plant disease recognition has emerged as a prominent agricultural trend. Quick, accurate, and effective identification of plant diseases and pests is crucial for improving crop yield and quality.

Researchers have proposed various techniques for detecting and classifying plant diseases in practical agricultural applications [3,4]. For example, [5] used pre-processing segmented lesion regions from diseased leaves. After completing feature extraction, they combined stepwise discriminant analysis, Bayesian discriminant analysis, principal component analysis, and Fisher discriminant analysis to classify strawberry disease leaves. The classification accuracy of this method reaches 94.71%. Choi et al. [6] classified five apple foliage diseases using eight features (color, texture, shape) with a BP neural network, which achieved a 92.6% average recognition accuracy. The above two methods adopt traditional manual feature extraction methods such as principal component analysis and texture feature extraction. Artificial feature extraction methods can achieve very good results on specific problems. But it also has obvious drawbacks such as requiring experts to invest a lot of time and effort in designing and adjusting features; the process is easily influenced by expert experience, and important features may be overlooked. These disadvantages have greatly limited the application of artificial feature extraction methods.

Deep-learning techniques have made remarkable advancements in plant disease recognition research in recent years. Dwivedi et al. [7] employed an attention mechanism within a residual module to effectively learn disease features and detect three diseases in tomato plants using the PlantVillage dataset. Notably, their approach achieved an outstanding detection accuracy of 98% across the three types of diseases. Luo et al. [8] used the residue network to identify maize diseases and used the TEL-Resnet network to identify and classify different leaves with pests and diseases. Ferentinos et al. [9] showcase GLDDN, a grape leaf disease detection network that utilizes dual attention mechanisms for feature evaluation, detection, and classification. Experimental results on the dataset demonstrate its superiority by achieving a 99.93% accuracy for three types of grape leaf disease detection. In the work of Gadekallu et al. [10], a hybrid-principal component analysis–whale optimization algorithm was employed to extract essential features from the dataset. These features were then input into a deep neural network for tomato disease classification, resulting in an accuracy of 94% for tomato disease recognition. Zhang et al. [11] proposed using a dual attention semantic segmentation network for corn recognition. The average cross-parallel ratio and average pixel recognition accuracy of the model are improved. In addition, deep learning generalization and regularization are also important methods for improving recognition accuracy [12]. Zheng et al. [13] used MobileViT, a lightweight neural network, to complete real-time automatic modulation classification. This method has strong robustness and high accuracy.

Traditional artificial feature extraction and deep learning-based methods are currently the most commonly used methods for plant disease recognition. They can achieve good results when applied to specific problems, but there are still some difficulties in practical applications: (1) The problem with training datasets. The performance of deep learning frameworks largely depends on the quality of a training dataset. High-quality datasets improve the accuracy and robustness of models by providing comprehensive and diverse data, thereby achieving better generalization and reducing bias in deep learning models. Collecting images and creating datasets require a significant amount of manpower and resources. (2) Noise interference problem. There are often many interfering factors in images taken from nature. These interference factors make it difficult to extract disease features from images. When training directly with these pictures, there are problems with learning disabilities and low recognition accuracy.

Therefore, in order to improve the speed and accuracy of plant disease identification, it is necessary to optimize existing identification methods. Image enhancement methods can solve the problems of training datasets and noise interference encountered during the recognition process. We can improve the accuracy of feature extraction by removing noise from images through image segmentation and enhancement. The dataset can also be expanded through methods such as rotation, brightness adjustment, and perspective transformation.

Images collected from nature commonly exhibit blurred features due to lighting issues. Therefore, before inputting images into the training model, the images must be processed. The processed image should have more prominent disease features to improve recognition accuracy. The Retinex method is the most classic image enhancement method, which enhances the contrast of images through logarithmic operations and Gaussian filtering [14]. On this basis, multi-scale analysis techniques are introduced to remove background noise. Wang et al. [15] proposed an underwater image enhancement framework based on metalantis. Their method is divided into three stages. This method first performs virtual underwater image synthesis and then estimates the depth map of the underwater image. Finally, reinforcement learning is used for underwater image enhancement. Jin et al. [16] introduced a new zero reference color self-calibration framework for enhancing low-light images. It effectively emphasizes the channel representation containing fine-grained color information, which achieves natural results in a progressive manner.

Currently, most researchers performing tomato plant disease classification only use image datasets from laboratory environments and do not include data from other domains. Moreover, the imbalanced distribution of samples in the dataset can hinder model generalization and accuracy, resulting in suboptimal performance on real-world test data. To address this issue, data augmentation techniques like flipping and rotation are extensively employed to enrich datasets and improve model robustness. However, when sample data are insufficient, traditional image enhancement techniques cannot generate new image features within a category. To enable models to learn new features from enhanced images, generative adversarial networks (GANs) have been proposed for application. The GANs still have many limitations such as the inability to generate changes for specific targets.

Ref. [17] used several basic image enhancement techniques, such as image rotation, brightness adjustment, perspective transformation, and affine transformation, to enhance their dataset and train the model to achieve better results. Using these base image augmentation techniques tends to generate highly correlated samples. These techniques are usually only applied to one image at a time, and overuse may lead to overfitting, making the enhanced image features lack variability.

The generative adversarial network (GAN) proposed by [18] has demonstrated remarkable capabilities in diverse image synthesis tasks. GANs unlock additional information about image features, resulting in more varied generated images and subsequently enhancing the performance of deep learning models. The primary learning goal of generative adversarial networks is to create synthetic samples with similar feature distributions to the training images.

Ref. [19] used DCGAN to increase the original dataset by two times to verify whether the GAN network could help improve the learning capabilities of the model. They trained on the InceptionV3 model, and they found that the accuracy of the enhanced dataset was improved by about 20%, compared with the original dataset. They show that GANsen-based data augmentation is beneficial for enhancing model learning performance.

Ref. [20] used Cycle-GAN to convert healthy apple images into diseased fruit images so that their proposed plant disease detection model improved by about 5% in the F1-score. Although Cycle-GAN has achieved excellent performance in image synthesis and style transfer, there is no mechanism to label specific objects in the image to be transformed, so the generated images may contribute little to the robustness of the model in plant disease diagnosis. This means that background regions that people are not interested in may also change together during this process.

In addition, some researchers have expanded the existing large datasets to alleviate the problem of insufficient data. Nevertheless, a model’s performance could be limited owing to the significant disparity between the collected natural environment pictures and the existing laboratory environment pictures and the notable distinction between the background environments in the field and laboratory settings [21].

Summarily, traditional image augmentation techniques lack feature changes and tend to generate highly correlated examples, which can lead to overfitting during training. However, with generative adversarial networks, existing studies are enhanced in the same category so that the new features after enhancement are limited. Based on Cycle-GAN, we introduce a segmentation algorithm so that the network can realize the transformation of specified regions of different categories of images. Thus, how to add as much obvious feature information as possible in images is a major challenge for image enhancement methods, and also a key problem for improving recognition accuracy.

In order to solve the above problems, we introduce a segmentation algorithm based on Cycle-GAN so that the network can realize the transformation of the specified region of different categories of images. Our study presents a deep learning framework for learning image features associated with tomato diseases through the training of a dataset that comprises tomato disease images. Through this approach, even in remote areas, farmers can easily capture leaf images that may be affected by diseases, using only mobile devices to determine the type of disease without relying on professional technicians. In our proposed method, we use techniques such as image augmentation, image segmentation, and image transformation to enhance the dataset and then train the pre-trained MobileNet [22] model on these enhanced datasets to separate multiple diseases from leaf images. Our contribution can be summarized as follows:

(1) This paper presents an automatic leaf segmentation algorithm (AISG) based on EISeg, which is designed to address the color characteristics of crop leaves in real-world environments. The AISG algorithm effectively removes environmental noise from leaves, enhancing the practical performance of CNN and Cycle-GAN networks.

(2) We introduce a leaf segmentation algorithm based on the Cycle-GAN network so that the network can realize the transformation of specific regions in the image. This image enhancement method realizes the transformation between different categories, enhances the image features, and has a particular application value.

(3) We collected crop images in the natural setting to augment the PlantVillage dataset, creating a new dataset. Transfer learning was employed for training the models. The capability of the method was evaluated through comparative experiments, thus validating its performance and potential for crop disease recognition.

The study is structured as follows: Section 2 introduces the image enhancement methods and the classification model system used. Section 3 provides details on the dataset, experimental design, result analysis, and comparisons with other methods. Section 4 presents the conclusion and future work. Section 4 is the conclusion and future work of this paper.

## 2. Methodology

**Figure 1:**
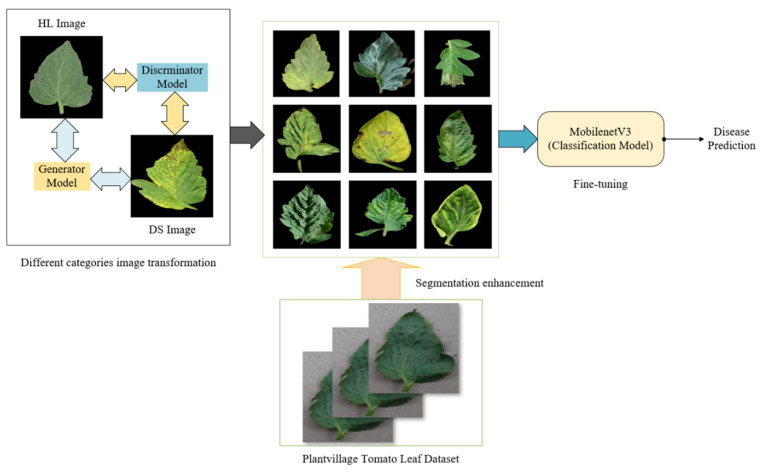
Framework of crop disease recognition system combining image processing and deep Learning.

We propose a cash crop disease recognition system to overcome the challenges arising from complex background compositions in crop leaf images taken in field environments, the diverse range of crops, and the uneven distribution of samples in existing disease datasets. The method proposed in this paper combines the advantages of image processing and deep learning techniques. This method can solve the constraints of complex backgrounds, uneven samples, and other factors, thereby achieving accurate disease identification. The flowchart of the proposed method is shown in Figure 1.

Our work can be divided into three main parts. Firstly, an automatic segmentation method of crop leaves based on the EISeg tool [23] was used to separate the disease leaves from the environmental background to facilitate the model in learning the significant features of the disease. Then, the Cycle-GAN network is used to realize the mutual transformation between different categories, and the small sample data are enhanced to improve the model’s generalization ability. Finally, the pre-trained MobileNet model was fine-tuned to realize the classification of crop diseases.

### 2.1. The AISG Algorithm

In the process of image acquisition in practical applications, there will inevitably be confusion about features of other objects in the images, such as the hands of the photographer, non-diseased leaves, soil, etc., which will significantly impact the classification results of the model. Hence, performing image segmentation is essential as it mitigates the impact of the objects above on the model’s discrimination, reduces computational overhead, and accelerates the classification process for each image. Based on the EISeg interactive segmentation method, aiming at its lack of human–computer interaction requirements, this paper proposes an improved EISeg interactive segmentation method.

**Figure 2:**
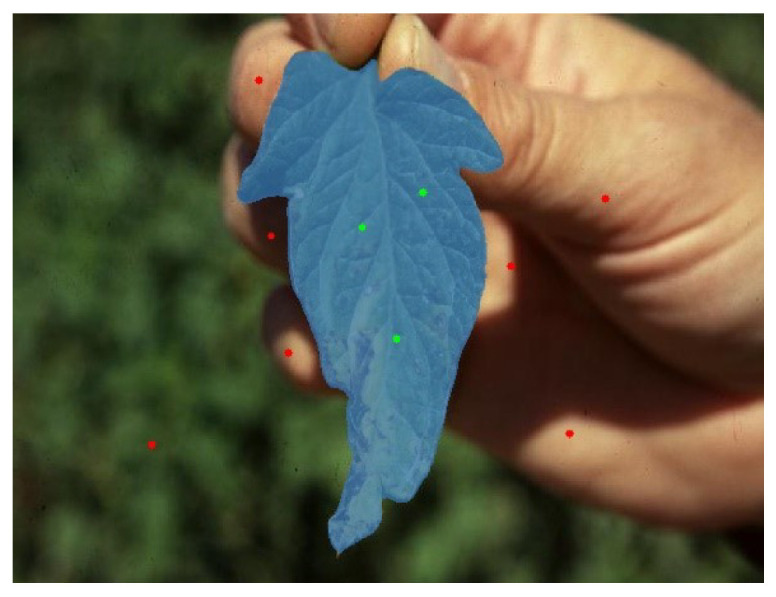
Illustration of interactive segmentation.

EISeg is a point-and-click interactive segmentation method. In practical applications, click-based methods commonly utilize positive and negative categories of user clicks. Positive clicks highlight the target object (foreground), while harmful clicks separate non-target regions (background). This method only requires a few clicks to complete the object segmentation task. Figure 2 is an example of this method. The EISeg algorithm has simple steps, but it requires someone to help click to complete the segmentation. So, the EISeg algorithm does not conform to the current trend, which greatly limits its application. The green dots in the picture represent positive clicks, indicating that the selected part is the foreground. The red dots represent negative clicks, indicating that the user has selected the background section.

**Figure 3:**
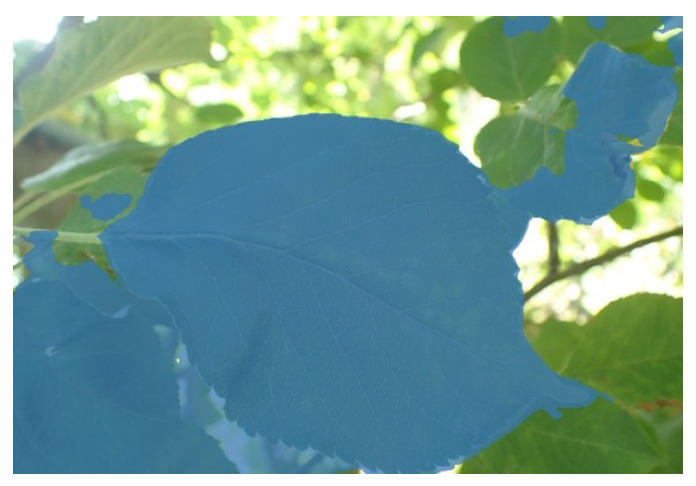
False segmentation.

Regarding this topic, we add some image processing steps before calling the EISeg model to make up for the lack of manual interaction of the EISeg model so that it can meet the needs of automatic, practical application. Before feeding the crop disease image into the model, we use the super-green factor to replace the manual calibration of the leaf and background pixels. For most crop leaves, the green component (G) in the color space (RGB) is much larger than the other components. According to this feature, during the initialization of the mask picture, the picture is processed by the green factor (2G-R-B), and the pixels whose super-green factor is inferior to the Border T are marked as the background. Otherwise, they are marked as the foreground. However, if only the super-green element is used for calibration, it will cause missegmentation when facing the background of many leaves, as shown in Figure 3.

**Figure 4:**
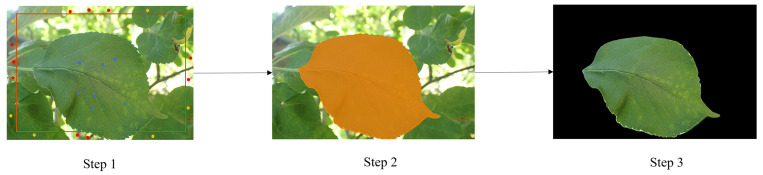
Schematic diagram of the AISG Algorithm.

Building upon the preceding analysis, this study will propose the following steps to enhance the segmentation method. The algorithm’s schematic diagram is depicted in Figure 4:

Step 1: Use the rectangle function of OpenCV to select the region of the target image. The rectangle’s dimensions are decreased by one-fourth of the original image’s side length, and an approximate plan for the target object’s location is devised.

Step 2: Using the super-green factor to determine the inside and outside of the rectangle, the green pixels outside the box are denoted as the possible background, and the non-green pixels are indicated as the background. In the inside of the rectangle, the points representing the foreground are marked according to the super-green factor to generate the mask map. The blue dots inside the rectangle indicate that the foreground is selected. The red and yellow dots outside the rectangle indicate that the background is selected. The yellow dots are green pixels, while the red dots are non green pixels.

Step 3: Call EISeg algorithm for image segmentation.

### 2.2. Cycle-GAN as Synthetic Image Generator

In deep learning model training, enough labeled data are needed to achieve better model performance. However, in practice, scarce or imbalanced data are common in the agricultural field, and labeled data are expensive or difficult to collect. Traditional image enhancement algorithms can only be varied within a specific category, but more desirable variations can improve performance [24]. Drawing upon the aforementioned analysis, our study will introduce a novel image enhancement method based on Cycle-GAN, which can realize the transformation from one category to another. The Cycle-GAN model inherits the idea of adversarial training of GAN and realizes the mapping between the source domain and the target domain without the pair relationship with the dual training and learning mode. This feature of Cycle-GAN allows it to migrate without paired datasets.

**Figure 5:**
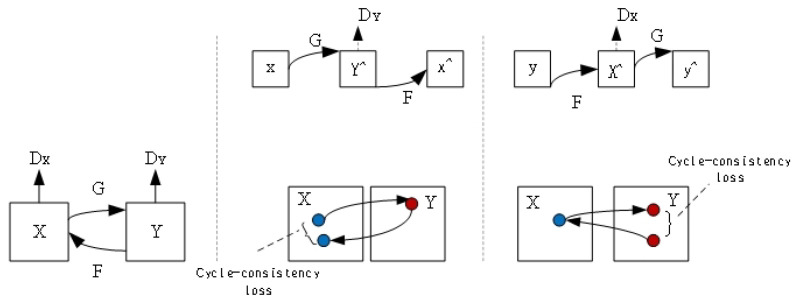
The network structure of Cycle-GAN.

Cycle-GAN is a generative adversarial network variant that maps images from one domain to another without matching the correspondence between the two domains. Specifically, Cycle-GAN works by splitting the mapping of a shot from one field to another into two mappings: One model facilitates the transformation from domain A to domain B, while the other enables the transformation from domain B to domain A. These mappings are designed to invert each other, so Cycle-GAN can ensure consistency and similarity by converting images to each other. The network structure of Cycle-GAN is shown in Figure 5. By training these mappings, Cycle-GAN can generate high-quality images, enabling many interesting applications such as style transfer, image conversion, and image enhancement.

Table 1 displays the architecture of the generator, while Table 2 presents the configuration of the discriminator in Cycle-GAN.

In this study, we found that the image transformation achieved by Cycle-GAN is often global. It will cause unnecessary background noise information to be transformed, which is harmful to the learning of the model. Therefore, before performing the Cycle-GAN transformation to generate different types of images, the image will be segmented once. The unwanted background will be removed in advance, to ensure the image transformation is carried out in the target area.

### 2.3. Disease Detection in Plant with MobileNet

MobileNet is a lightweight, efficient convolutional model that is ideal for mobile devices. Therefore, we chose MobileNet as the training model. Incorporating pre-trained MobileNet, a lightweight convolutional network trained on the ImageNet dataset, for tomato crop disease recognition through the transfer learning technique.The MobileNet architecture used is described as follows:

MobileNet has undergone three generations of updates, and MobileNetv1 uses depthwise separable convolutions to build lightweight networks [25]. MobileNetV2 introduces a novel inverted residual with a linear bottleneck unit, resulting in improved network accuracy and speed despite the increased number of layers. MobileNetV3 takes advantage of both machine learning techniques and manual fine-tuning to construct a more efficient and lightweight network.

The model used in this paper is MobileNetv3, and the description of each layer is shown in the Table 3. It comprises 15 Bneck layers, one standard convolutional layer, and three pointwise convolutional layers, taking 224 × 224-pixel images as input. The first convolutional layer unrolls the 2242 × 3 image input, and the middle is 15 Bneck layers to learn the image features. It is followed by a pooling layer and two BN convolutional layers. Each Bneck contains two pointwise convolutional layers and one deep convolutional layer.

## 3. Implementation and Results

### 3.1. Dataset Description

The PlantVillage dataset, developed by David Hughes and Marcel Salathe, is a widely used publicly available database and holds a prominent position in the field. It contains 38 categories for 14 plants and 26 diseases and 54,036 images. It is very suitable for training plant disease detection and recognition models. However, the images in this dataset are taken under a single background in a laboratory environment, there are no images taken under complex natural conditions, and the number of samples is not balanced.

In order to ensure a balanced sample size in the dataset and a closer approximation to real field scenes. We constructed our initial dataset through three different approaches. Firstly, we selected 8000 laboratory environment photos from the PlantVillage dataset. Then, we obtained 1824 photos of outdoor environments from various search engines. In addition, we utilized local planting bases and obtained 1892 images of tomato diseases in complex environments under the guidance of professionals. Therefore, the initial dataset consists of 11,716 photos. The sample size in the initial dataset is unbalanced and insufficient to support achieving good recognition accuracy. Based on this, we have made certain extensions to the dataset. Firstly, the sample images were subjected to horizontal, vertical, random, and reverse folding operations, resulting in an additional 3502 images. Then, we generated 3100 images using Cycle-GAN. The expanded dataset contains a total of 18,318 images. We classified various disease states of tomato crops into a compact dataset and further divided it into training, validation, and testing sets in an 8:1:1 ratio. The detailed distribution of the images is shown in Table 4.

### 3.2. Experimental Configuration and Evaluation Criteria

In the experiment, we used the expanded dataset for training and testing. We use the PyTorch deep learning framework, and the programming environment is Python 3.6. Referring to relevant literature, we set the PyTorch hyperparameters: Batch Size, Epochs, and Learning Rate [26]. The Batch Size is 64, the Epoch is 20, and the Learning Rate is 0.001. The other experimental configurations are Windows 10 (64-bit) operating system, 16GB of experimental platform memory, Intel Core I7-9700K CPU with NVIDIA GeForce GTX1660Ti 6GB GPU(GPU sourced from NVIDIA, Santa Clara, CA, USA).

This study comprises five sets of experiments. The initial group of experiments evaluates the test images using the segmentation algorithm before and after enhancement to assess the algorithm’s effectiveness. In the second group of experiments, the trained Cycle-GAN is used to enhance the small sample data, and the performance of Cycle-GAN is qualitatively analyzed from the transformation effect, the quality of the generated image, and the way of control experiments. In the third set of experiments, we divide the dataset into four different types to train the model separately to verify the impact of data augmentation on the model’s performance. The fourth set of experiments compared our method with the methods adopted by other researchers. The fifth set of experiments is a generalization experiment to verify the recognition accuracy of the proposed method on other plant leaves.

Following the experiments, the proposed method’s performance was assessed using various metrics, including *Acc*, *Pre*, *Rec*, *F*1-*score*, and the confusion matrix.

(1) $$\begin{array}{c} Acc=\frac{TP+TN}{TP+TN+FP+FN} \end{array}$$

(2) $$\begin{array}{c} Pre=\frac{TP}{TP+FP} \end{array}$$

(3) $$\begin{array}{c} Rec=\frac{TP}{TP+FN} \end{array}$$

(4) $$F1-score=\frac{2\times TP}{2\times TP+FP+FN}$$

Here, the notation *TP* represents True Positive, *TN* stands for True Negative, *FP* denotes False Positive, and *FN* represents False Negative.

Furthermore, the Peak Signal-to-Noise Ratio (*PSNR*), an image quality metric, was employed to assess the quality of synthetic pictures generated by Cycle-GAN.

(5) $$\begin{array}{c} PSNR=20.\log_{10}\left(MAX_{I}\right)-10.\log_{10}\left(MSE\right) \end{array}$$

(6) $$MSE=\frac{1}{mn}\sum_{0}^{m-1}\sum_{0}^{n-1}{\left[I\left(i,j\right)-K\left(i,j\right)\right]}^{2}$$

where $MAX_{I}$ represents the maximum possible pixel value in image *I*, and *MSE* denotes the mean squared error.

### 3.3. Result and Performance Analysis

This section presents the performance evaluation of the proposed method.

#### 3.3.1. Performance of AISG Algorithm

**Figure 6:**
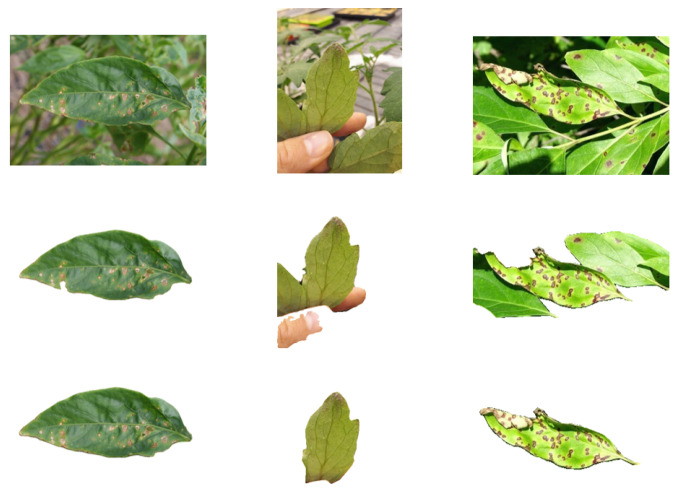
Diagram of the segmentation effect.

Within this section, we evaluate the effectiveness of the AISG method proposed in this study by segmenting three randomly chosen images from the dataset and conducting a comprehensive analysis. Figure 6 compares segmentation results between AISG and EISeg algorithms on sample data. The first row of the image represents the original image, the second row represents the image segmented by the EISeg algorithm, and the third row represents the result of our AISG algorithm. Comparing the experimental results of the second and third rows, we can see that the optimized AISG algorithm can better remove background noise while preserving leaf information. From the second and third columns of the figure, it can be seen that when there are multiple leaves or the lesion site overlaps with the leaves, the EISeg algorithm cannot preserve the lesion information well. And our AISG algorithm is able to separate target leaves with lesion information included. The results in Figure 6 indicate that the AISG algorithm has better performance.

In this study, we evaluate the efficiency of the two algorithms through extensive experiments and adopt the average time as the evaluation metric. In order to avoid errors and improve the effectiveness of the results, we segmented the images of the dataset five times and verified them in the actual scene image set. We measured the segmentation time and accuracy of the two algorithms for each image. The results in Table 5 demonstrate that the AISG algorithm outperforms the EISeg algorithm, exhibiting a remarkable 7% improvement in accuracy. Moreover, the AISG algorithm reduces approximately 3 s in the average time required for image segmentation. The enhanced stability of segmentation results further validates the effectiveness of the AISG algorithm, highlighting its significant efficiency improvement in image segmentation.

#### 3.3.2. Performance of Cycle-GAN

**Figure 7:**
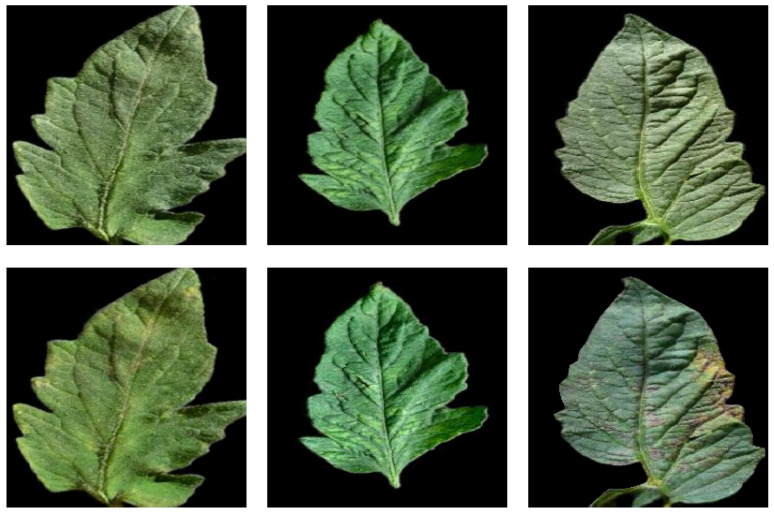
The GAN generation effect.

In this section, we conduct a qualitative analysis of the Cycle-GAN performance. Figure 7 displays tomato leaf pictures from the augmented dataset and the corresponding diseased tomato leaf pictures transformed by Cycle-GAN. As shown in the figure, the first row of images is the original image in the dataset, and the second row displays the transformed image. From the first and second columns of images, it can be seen that the synthesized image is very similar to the original image. This indicates that our transformation method has good performance. The two images displayed in the third column have similar structures, but there are significant differences in color. This is because the lesion features are hidden in the original image due to lighting conditions. After the Cycle-GAN transformation, the feature information in the image becomes more prominent. Furthermore, we compare the PSNR values of the initial images with the transformed synthetic images to assess the difference in image quality. Table 6 presents the PSNR values, revealing that the images generated by the Cycle-GAN transformation closely resemble the quality of the original images. To further evaluate the quality of the images after Cycle-GAN transformation, we compared the multi-scale structural similarity (MS-SSIM) of the images. Table 7 presents the MS-SSIM values, indicating a high degree of structural similarity between the images generated by Cycle-GAN transformation and the original images.

**Figure 8:**
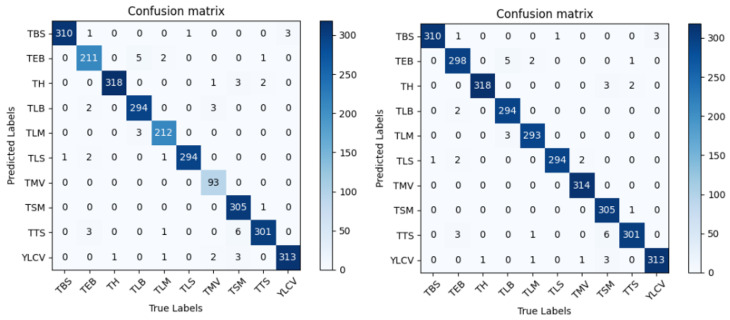
Identification result confusion matrix.

At the same time, we also designed a set of comparative experiments to verify the influence of generated images on the model’s generalization performance. In the experiment, we trained the segmented dataset without small sample data augmentation and the segmented dataset with small sample data augmentation. Their confusion matrices are shown in Figure 8. The accuracy of several diseases enhanced by small sample data is improved by about 1%, which shows that the model’s generalization can be enhanced by using Cycle-GAN to generate small sample data.

#### 3.3.3. Performance of Classification Model

In this section, we divide our dataset into the following four categories:

Data Model A ( DMA): Train the MobileNet model using the PlantVailage dataset without any processing. The resulting model is named DMA.

Data Model B ( DMB): The crop disease data in the field environment were added to the PlantVailage dataset, and the MobileNet model trained on this basis was named DMB.

Data Model C ( DMC): The extended PlantVailage dataset was segmented and enhanced before being input into the MobileNet model for training. The resulting model is named DMC.

Data Model D ( DMD): The extended PlantVailage dataset is enhanced with small sample data and then input into the MobileNet model for training after segmentation and enhancement. The resulting model is named DMD.

Based on this foundation, we assess the classification performance of the proposed model on four datasets: DMA, DMB, DMC, and DMD. The experimental results are presented in Table 8. And the classification accuracy of our method in the four dataset classification tasks is 95.74%, 93.8%, 98.18%, and 98.61%, respectively. Comparing DMA with DMB, we can find that adding images in the natural environment for training will cause a particular decline in the model’s classification accuracy. Therefore, we added a set of tests and input 20 field images into DMA and DMB, respectively, for classification experiments. In the classification experiment, the accuracy of DMB reaches 85%. In comparison, the accuracy of DMA is only 70%, which proves that the use of natural environment images for training is more suitable for practical applications. When comparing DMB with DMC, it becomes evident that removing background noise through segmentation processing can enhance the model’s accuracy. Table 8 illustrates that the DMD model, implemented with the proposed method, exhibits improvements in acc, pre, rec, and F1-score across all categories when contrasted with the initial dataset. Furthermore, Table 9 showcases the classification capability of the proposed method on a 10-class classification task using the augmented dataset containing synthetic images.

In order to avoid the influence of accidental errors on the experimental results, we used k rounds of cross validation to further evaluate the performance of the model. The Fold cross validation method can effectively verify the recognition accuracy of the model. The principle of Fold cross validation is to divide the raw data into groups and calculate them separately. Therefore, the results calculated by this method are more convincing. In this experiment, we used 10-fold cross validation, which means taking the average of 10 results as the final data. The experimental results are shown in Table 10.

#### 3.3.4. Performance of Comparison

Compared to other convolutional networks, MobileNet has the characteristics of being lightweight and efficient. It reduces the complexity of the network by reducing the number of linked or non-fully connected layers, decreasing the number of channels and convolution kernel size, removing some feature maps, pruning and discretizing some unimportant weights, etc. The MobileNetV3 used in this article combines the advantages of MobileNetV1 and MobileNetV2 with faster speed and accuracy. In addition, MobileNet performs well in application scenarios on mobile devices. And our subsequent research is to apply our method to mobile devices, so we chose MobileNet as the training model.

The fine-tuned MobileNet model was compared with other CNN networks in classification experiments. The performance of the models is depicted in the Table 11. All models are individually trained using the DMA and DMD methods. Notably, the MobileNet model stands out by achieving an outstanding accuracy rate of 98.61% when tested on the augmented dataset, surpassing the performance of all other pre-trained models. The results evidently demonstrate that the expanded dataset, incorporating the methods employed in this study, yields superior accuracy compared to the original dataset.

Numerous researchers have previously introduced diverse methods for detecting tomato plant diseases in tomato leaf images. In this study, we compare our method with other approaches presented in past research based on the PlantVillage dataset. Table 12 provides an overview of the effectiveness comparison between the proposed method and existing approaches. The research detailed in the table primarily focuses on the classification task involving 10 categories of tomato diseases.

In this experiment, we compared the experimental results of the proposed method with other algorithms. The compared algorithms include the classic CNN model, ResNet101, and other advanced algorithms (HCA MFFNet, ICAI-V4). As shown in Table 12, our model outperforms the compared algorithms in F1, accuracy, recall, and average accuracy metrics. In terms of training time, this model also has the shortest training time. This indicates that our method has better performance.

#### 3.3.5. Generalization Experiment

Generalization ability is an important indicator for evaluating the practicality of a model. If a model can be applied to different types of plant leaf datasets, then the model has strong generalization ability. The applicability and scalability of the proposed method can be verified through generalization experiments. In the generalization experiment, we applied the proposed method to different datasets to compare accuracy and recall. The dataset used in this experiment mainly comes from two public datasets: the PlantVillage and ImageNetDogs datasets. Both datasets contain laboratory images and field images. We chose images of grapes, tobacco leaves, corn, and apples from PlantVillage for the experiment.

The recognition accuracy of our model on different datasets is shown in Table 13. Our model has high recognition accuracy for different types of plant disease images. According to the experimental results, it can be seen that the method proposed in this paper can be applied to different datasets and has good generalization ability.

## 4. Conclusions

In recent times, deep learning-based approaches have exhibited remarkable advancements in crop disease detection. Training datasets and noise interference seriously affect detection accuracy. To solve this problem, we propose a method that combines image processing and deep learning. In the proposed method, we expand the dataset in the laboratory environment to make it more suitable for practical applications. Then, the background noise is removed by image segmentation to improve the model’s accuracy and prevent the Cycle-GAN network from translating unnecessary background information together. Then, the small sample data are enhanced by the Cycle-GAN network. Finally, the enhanced dataset is input into a fine-tuned moblienet for training. The proposed data augmentation method improves the model’s accuracy, enhances the network’s generalization, and avoids the overfitting problem. Our proposed method obtained an accuracy of 95.74% on the original PlantVillage dataset, which significantly improved to 98.61% after applying the augmented dataset. The experimental results show that our method is superior to existing methods. At the same time, the proposed algorithm is not only applied to the field of tomato leaf diseases but can also detect and identify other crop diseases. The proposed algorithm is of great value for crop yield and crop pest control.

The limitation of our research is that we did not consider the issue of two or more diseases appearing on one image. This situation is likely to occur in practical applications, and our method still needs to improve the accuracy of identifying multi-disease leaves. Therefore, in future research, we will further optimize our model to make it applicable to more complex real-world scenarios. Another future research direction is to integrate this disease recognition and classification method into mobile terminals, thus providing a practical and powerful tool for the development of smart agriculture. 

## Figures and Tables

**Table 1:** The structure of the generator network for Cycle-GAN.

Layer	Tpye	Parameter
lnput	lnput	3 × 256 × 256
Reflection Padding	conv	3 × 336 × 336
32 × 9 × 9 conv, stride 1	conv	32 × 336 × 336
64 × 3 × 3 conv, stride 2	conv	64 × 168 × 168
128 × 3 × 3 conv, stride 2	conv	128 × 84 × 84
Residual block, 128 filters	Residual block	128 × 80 × 80
Residual block, 128 filters	Residual block	128 × 76 × 76
Residual block, 128 filters	Residual block	128 × 72 × 72
Residual block, 128 filters	Residual block, 128 filters	128 × 68 × 68
Residual block, 128 filters	Residual block, 128 filters	128 × 68 × 68
64 × 3 × 3 conv,stride 1/2	conv	65 × 128 × 128
32 × 3 × 3 conv,stride 1/2	conv	32 × 256 × 256
3 × 9 × 9 conv,stride 1	conv	3 × 256 × 256

**Table 2:** The discriminator Network Architecture of Cycle-GAN.

Layer	Tpye	Parameter
lnput	lnput	3 × 256 × 256
Down block	Conv2d	4 × 4, 64
Down block	Conv2d	4 × 4, 128
Down block	Conv2d	4 × 4, 256
Down block	Conv2d	4 × 4, 512
Conv, stride 1	Conv2d	4 × 4, 1
output	output	16 × 16 × 1

**Table 3:** The structure of MobileNet.

Layer	Input	Output	RELU
Conv2d	2242 × 3	16	HS
Bneck, 3 × 3	1122 × 16	16	RE
Bneck, 3 × 3	1122 × 16	24	RE
Bneck, 3 × 3	562 × 24	24	RE
Bneck, 5 × 5	562 × 24	40	RE
Bneck, 5 × 5	282 × 40	40	RE
Bneck, 5 × 5	282 × 40	40	RE
Bneck, 3 × 3	282 × 40	80	HS
Bneck, 3 × 3	142 × 80	80	HS
Bneck, 3 × 3	142 × 80	80	HS
Bneck, 3 × 3	142 × 80	80	HS
Bneck, 3 × 3	142 × 80	112	HS
Bneck, 3 × 3	142 × 112	112	HS
Bneck, 5 × 5	142 × 112	160	HS
Bneck, 5 × 5	72 × 160	160	HS
Bneck, 5 × 5	72 × 160	160	HS
Conv2d, 1 × 1	72 × 160	960	HS
Pool, 7 × 7	72 × 960	-	-
Conv2d 1 × 1, NBN	12 × 960	1280	HS
Conv2d 1 × 1, NBN	12 × 960	k	-

**Table 4:** The details of dataset.

Category	Sum	Laboratory	Field	GAN
Bacterial Spot (TBS)	1587	1031	256	300
Early Blight (TEB)	1564	900	164	500
Healthy (TH)	1637	1637	0	0
Late Blight (TLB)	1556	1142	214	200
Leaf Mold (TLM)	1508	981	127	400
Leaf Spot (TLS)	1493	1000	293	200
Target Spot (TTS)	1571	1200	271	100
Spider Mites (TSM)	1628	1100	228	300
Mosaic Virus (TMV)	1458	430	128	900
Yellow leaf curl virus (YLCV)	1606	1128	278	200

**Table 5:** The correct rate and average time of segmentation algorithm.

Method	Correct Rate	Average Time (s)
EISeg	88.5%	4.01 s
AISG	95.3%	0.83 s

**Table 6:** The PSNR values are calculated between the original disease images and the Cycle-GAN generated images for three classes.

Category	OR (dB)	GE (dB)
Leaf Mold (TLM)	30.23	31.367
Mosaic Virus (TMV)	31.114	32.944
Early Blight (TEB)	33.273	35.573

**Table 7:** The MS-SSIM values are calculated between the original disease images and the Cycle-GAN generated images for three classes.

Category	CC	ROCC
Leaf Mold (TLM)	0.971	0.969
Mosaic Virus (TMV)	0.978	0.981
Early Blight (TEB)	0.975	0.977

**Table 8:** Acc, pre, rec, and F1-score of the models trained on different datasets.

Model	Acc	Pre	Rec	F1-Score
DMA	0.9574	0.9578	0.9574	0.9852
DMB	0.938	0.94	0.9378	0.9831
DMC	0.9818	0.9827	0.9784	0.9802
DMD	0.9861	0.9861	0.9857	0.9859

**Table 9:** Pre, Rec, and F1-score for ten disease classes of tomato.

Class	Pre	Rec	F1-Score
TBS	0.984	0.997	0.99
TEB	0.98	0.974	0.977
TH	0.985	0.995	0.992
TLB	0.993	0.974	0.983
TLM	0.99	0.99	0.99
TLS	0.983	0.997	0.99
TMV	1	0.991	0.995
TSM	0.997	0.962	0.979
TTS	0.965	0.987	0.976
YLCV	0.984	0.99	0.987
mean	0.9861	0.9857	0.9859

**Table 10:** Ten-fold cross validation.

Fold	Accuracy
1	91.5%
2	95.3%
3	97.8%
4	95.1%
5	94.5%
6	97.4%
7	89.2%
8	90.7%
9	96.5%
10	95.8%

**Table 11:** Performance of other models on the augmented dataset.

Model	Plant Village	Augmented Dataset
VGG16	86.71%	88.62%
Resnet50	76.82%	81.15%
Inception	82.13%	85.42%
MobileNetv3	95.74%	98.55%

**Table 12:** Comparison with other methods.

Model	F1 (%)	Acc (%)	Pre (%)	Rec (%)	Time
[27]	89.25	89.63	88.76	89.58	413.26
[28]	90.85	90.76	90.28	90.65	428.67
[29]	92.85	93.36	93.05	93.23	419.21
[30]	94.23	94.33	93.85	94.02	415.32
Our model	97.03	96.89	96.96	97.11	393.51

**Table 13:** Results of different datasets.

Dataset	Acc (%)	Rec (%)
ImageNetDogs	98.17	98.02
PlantVillage grape	98.35	98.23
PlantVillage tobacco	98.56	98.59
PlantVillage maize	98.67	98.54
PlantVillage apple	97.89	97.81

## Data Availability

The raw data supporting the conclusions of this article may be provided upon reasonable requests for scientific research purposes.
